# Spontaneous tension pneumomediastinum with pneumothorax and subcutaneous emphysema as a complication of COVID‐19 disease

**DOI:** 10.1002/ccr3.7570

**Published:** 2023-07-10

**Authors:** Manouchehr Aghajanzadeh, Ali Alavi Foumani, Azita Tangestaninejad, Mohammad Haghighi, Yousha Pourahmadi, Ehsan Hajipour Jafroudi, Mahsa Mousazadeh, Rastin Hosseinzadeh Asli

**Affiliations:** ^1^ Department of Thoracic Surgery Guilan University of Medical Sciences Rasht Iran; ^2^ Department of Pulmonology Guilan University of Medical Sciences Rasht Iran; ^3^ Department of Anesthesiology Guilan University of Medical Sciences Rasht Iran; ^4^ Department of Internal Medicine, Inflammatory Lung Diseases Research Center Guilan University of Medical Sciences Rasht Iran

**Keywords:** COVID‐19, pneumothorax, spontaneous tension pneumomediastinum, subcutaneous emphysema

## Abstract

Recently spontaneous tension pneumomediastinum (STM), were reported as infrequent complications in coronavirus disease 2019 (COVID‐19) patients but pneumothorax (PT), and subcutaneous emphysema (SE) are more frequently seen in COVID‐19 patients. PT and SE may present after PTM in COVID‐19. The aim of this presentation is to show the complications of STM in an Iranian patients with COVID‐19 disease with PT and SE, who were hospitalized in Arya hospital, Rasht, Iran. For 3 months, we followed these patients and their condition was good. STM are uncommon complications in COVID‐19 patients and were reported frequently in male patients. Early diagnosis and treatment could save the patients as these complications are related to poor prognosis and prolonged hospitalization. Patients with mild COVID‐19 and mild pulmonary damage may have a favorable outcome.

## 1 INTRODUCTION

Severe acute respiratory syndrome coronavirus (COVID‐19) infection has developed as the greatest pandemic of the world with high morbidity, mortality, and several complications. There are lots of studies about (COVID‐19) and its complications.^1^, ^2^, ^3^ There are reports about spontaneous pneumomediastinum (PM), pneumopericardium (PP), pneumothorax (PT), and subcutaneous emphysema (SE) cases in patients with COVID‐19, and also some of these complications present in the patients with mechanical ventilation, which results in barotrauma.^4^, ^5^, ^6^ The pathophysiologic mechanism of PM is an increased gradient pressure between the alveoli and parenchyma of lung that leads to severe alveolar injury and the air dissects the surrounding bronchovascular sheaths, and this air enters into the mediastinum and produces PM and disturbance to cervical SE and pleural space PT.^2^, ^4^ The pulmonary complications are a sequela of parenchymal, vascular, or pleural involvement and range from pneumonia to acute respiratory distress syndrome (ARDS) and pulmonary embolism to PT.^7^ PT, defined as air within the pleural space, is one of the emerging complications of COVID‐19 infection.^8^ Alveolar wall is more prone to rupture due to inflammation in COVID‐19 patients and alveolar wall is exacerbated by severe cough or any problems which increase the intra‐alveolar pressure.^4^ Invasive positive pressure ventilation can cause PT, SE, PP, and PM spontaneously, But spontaneous tension pneumopericardium or spontaneous pneumothorax tension is rare complication in COVID‐19.^9^ Barotrauma is the cause of PM, PT, PP, and SE.^2^ In this case report, we focus only on hospitalized COVID‐19 patients who developed these complications and diagnosis and treatment.

## 2 CASE PRESENTATION

**FIGURE 1:**
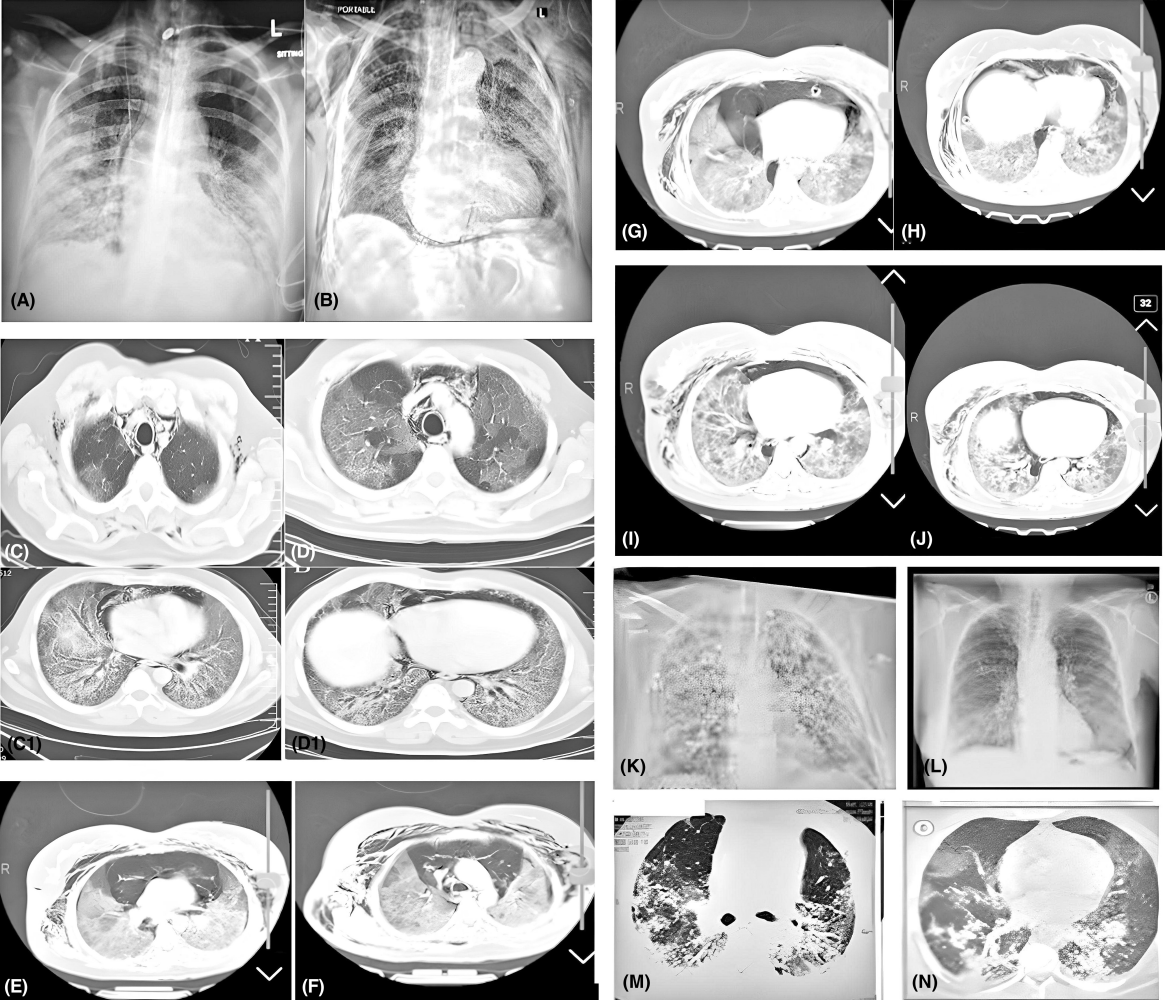
(A,B) Chest x‐ray on presentation with COVID‐19 pneumonia showing bilateral middle, upper, and lower zone infiltrates. (C,D and C1, D1) CT‐scan showing development of right and left side‐milled PT, PM. (E,F) CT‐scan of chest show tension pneumomediastinum, extensive SE and PT with compress of heart and mediastinum. (G,H) CT‐scan of chest show decreased the size of mediastinum and SE after chest tube and subclavicular incision. (I,J) CT‐scan of chest show decreased the size of mediastinum and SE after chest tube and subclavicular incision. (K,L) show CXR post 3 months after treatment. (M,N) CT‐scan of chest show post COVID 3 months after treatment.

A 35‐year‐old Iranian man presented with a 2‐day history of sore throat and mild shortness of breath. There was no history of fever or chest pain. The patient did not have any history of suspicious contact with COVID‐19 patients. He did not have any chronic medical illnesses, and was a non‐smoker. On physical examination, he had a temperature of 37.5°C, blood pressure of 130/82 mmHg, and respiratory rate (RR) of 26 breaths/min. His oxygen saturation on room air was 82% and required 10 liters of oxygen via a face mask to maintain oxygen saturation above 95%. The patient had laryngeal erythema and on respiratory examination bilateral coarse crackles was heard. Complete blood count revealed lymphopenia but others were normal (WBC, hemoglobin, and platelet counts). Alanine aminotransferase (ALT), aspartate aminotransferase (AST), and lactate dehydrogenase were elevate C‐reactive protein (CRP) and interleukin‐6 (IL‐6) were elevated, but d‐dimer level was normal (Table 1). A chest x‐ray and CT‐scan showed bilateral middle and lower and upper zone infiltrates (Figure 1A–D). A RT‐PCR nasopharyngeal swab was positive for SARS‐CoV‐2.

**TABLE 1:** Laboratory parameters of the patient at admission.

Parameter (normal range)	Results
White cells (4–10 × 10^3^/mm^3^)	6.8
Neutrophil (2–7 × 10^3^/mm^3^)	3.7
Lymphocyte (1–3 × 10^3^/mm^3^)	0.7
Platelets (150–400 × 10^3^/mm^3^)	185
Hemoglobin (12.5–13.5 gm/dL)	12.6
Red Blood cells (4.5–5.5 × 10^3^/mm^3^)	457
ALT (0–55 Unit/L)	269
AST (5–34 Unit/L)	97
Sodium (mmol/L)	138
Potassium (mmol/L)	4.3
D‐dimer (<mg/L FEU)	0.56
CRP (0–5 mg/L)	83.1
Procalcitonin (<0.5 ng/mL)	0.36
Ferritin (48–420 μgm/L)	1031
Interleukin−6 (≤7 pg/mL)	37

Abbreviations: ALT, alanine aminotransferase; AST, aspartate aminotransferase; CRP, C‐reactive protein.

The patient was admitted to the intensive care unit as a case of severe COVID‐19 pneumonia. As per the institutional protocol at the time, he was treated with intravenous (IV) ceftriaxone, azithromycin, methylprednisolone (40 mg every 12 hourly), and IV remdesivir. Despite treatment, the patient's condition did not improve. The patient received multidisciplinary care throughout his admission, including physiotherapy, occupational therapy, respiratory therapy, critical care, and infectious disease. On Day 8 of ICU stay, due to persistently high oxygen requirements and increasing D‐dimers (peak 8 mg/L FEU), a CT pulmonary angiogram (CTPA) was performed to rule out pulmonary embolism. The scan showed no filling defect but showed predominantly peripheral and patchy basal areas of ground‐glass attenuation with multifocal segmental dense consolidation with air bronchograms, consistent with severe bilateral pneumonia due to COVID‐19. In this situation O_2_ saturation and blood pressure dropped and CT‐scan of chest performed and show tension pneumopericadium (Figure 1E,F). patient as soon as possible referred to ICU with CPAP and bilateral chest tube was insert in third intercostal space in mid clavicular line and bilateral subclavicular incision for evacuation of subcutaneous air the condition of patient improved over the next 8 days, the patient's pneumeditiastinum moderately improved with decreased oxygen requirement (Figure 1G,H,I,J). However, due to persistent shortness of breath, oxygen requirement, and persistent bubbling of chest tube, pleurodesis with autologous blood and providon iodine^8^, ^10^ the air bubbling was not stopped. The patient's chest tube could not be removed till 2 months later when his pneumediastinum resolved, and he started to maintain normal oxygen saturation in room air. He was discharged after ward in an asymptomatic condition with bilateral chest rube which connected to urine bag and 45 days chest tubes was removed with no evidence of pneumeditiastinum and PT recurrence during 4 months following (Figure 1K–N) but patchy infiltration not improved.

## 3 DISCUSSION

The COVID‐19, has caused over 300 million cases and more than 5.4 million deaths globally since 2019.^1^, ^2^ Although most cases of COVID‐19 infection exhibit primarily constitutional and respiratory tract symptoms (such as fever, fatigue, myalgias, dry or productive cough, and dyspnea) similar to any other pulmonary viral infection.^1^, ^2^ Concerning the pulmonary manifestations of COVID‐19, other than pneumonia and ARDS, various complications have been reported, which are not routinely seen in other types of respiratory viral infections.^1^, ^2^, ^3^ These include a prolonged infectious state, lung fibrosis, bullous lung disease, pleural effusion, pulmonary cysts, spontaneous pneumothorax, and PM amongst others.^11^, ^12^ Most of the patients who die from COVID‐19 infection have the respiratory system as the primary organ involved.^13^

Tension pneumomediastinum is a rare but potentially lethal condition seen in critically ill patients. Traditionally, PM occurs in young patients with asthma.^1^, ^10^ During an asthmatic attack, rapid breathing causes alveolar rupture into the lower‐pressure mediastinum. This condition is often harmless and resolves spontaneously as air is absorbed with time.^3^, ^10^ On the other hand, tension pneumomediastinum can also occur due to prolonged mechanical ventilation, particularly in settings of high end‐expiratory pressure.^14^ Understandably so, this complication has seen an increase in incidence following the emergence of the COVID‐19 pandemic, as high end‐expiratory pressure ventilation has been utilized to a greater extent for management of COVID‐19–related respiratory distress.^1^, ^10^ This form of PM is far more complicated and requires urgent intervention.^10^

Tension pneumomediastinum is thought to occur in patients with COVID‐19 secondary to diffuse alveolar damage^10^, ^15^ as our patient. The increased presence of diseased alveoli on the mediastinal surface allows for preferential rupture into the mediastinum due to the pressure gradient between the alveoli and the perivascular sheaths.^10^ Further spreading of the pulmonary interstitial emphysema into the mediastinum is subsequently known as the Macklin effect.^12^ In patients with COVID‐19, the diseased lung may create a one‐way valve at the mediastinal pleural border, which can subsequently lead to air retention in the mediastinum.^10^ Increased pressure in the mediastinum can compress mediastinal contents. In particular, compression of the great vessels can lead to decreased venous return, hypotension with tachycardia, and potential cardiovascular collapse.^10^, ^12^, ^16^

Currently, management for tension pneumomediastinum in the COVID‐19 population has largely been conservative.^14^, ^17^ Different approaches include reducing airway pressures and adjusting ventilator settings to allow for permissive hypercapnia in an effort to reduce pressure gradients across the mediastinal surface.^3^, ^17^ These methods may be sufficient for the management of tension pneumomediastinum in stable COVID‐19 patients,^10^, ^17^ but those who are unstable may require immediate surgical decompression. After review of the current literature, Some describe cases report of operative management for a massive tension pneumomediastinum secondary to COVID‐19.^14^, ^16^, ^17^ Of note, there was some previous report of tension pneumomediastinum secondary to COVID‐19 that resolved with bedside mediastinotomy via the Chamberlain procedure.^10^, ^16^, ^17^, ^18^

In our patient with COVID‐19, a tension pneumomediastinum formed in the chest and SE in the neck, with subsequent spread to the arms bilaterally and with the enlarging PM caused difficulty breathing and progressive dysphonia with an increased pitch in the tone of his voice and engorge the jugular vein with cyanosis of face. Due to impending airway obstruction, the patient was sent for emergent mediastinal drainage with bilaterally chest tube insertion in anterior mediastinum and bilaterally sub‐clavicular incision for evacuation of SE. In some report they created a subxiphoid pericardial window, employed subxiphoid and suprasternal drainage of the PM, and performed substernal dissection with lighted scope^10^, ^16^, ^17^, ^18^ but we used bilaterally chest tube insertion in the anterior mediastinum. With these surgical managements, the anterior mediastinum was decompressed, resulting in rapidly reduced swelling in the patient's neck, improvement of his voice, and disappearance of the crepitus with clinical and radiographic healing but chest tube insertion is simple and available in emergency room or intensive care unit.

We describe the first minor invasive operative management of massive tension pneumomediastinum secondary to COVID‐19 infection. We used chest tube insertion that provided rapid decompression of unstable tension pneumomediastinum with mediastinal drainage. This case demonstrates that precipitous decline may occur in a patient with diseased lung parenchyma such as COVID‐19, and that our method may offer an effective operative solution for rapid decompression required for massive tension pneumomediastinum and SE.

## 4 CONCLUSION

Spontaneous tension pneumothorax should be included in the differential diagnosis of COVID‐19 infected patients, especially when worsening symptoms or develop new respiratory symptoms during their hospital course. The management should be the same as any spontaneous tension pneumothorax and emergency drainage of mediastinum is lifesaving; however, an early diagnosis and prompt treatment may help reduce the higher expected mortality with this approach in complication of COVID‐19 infection.

## AUTHOR CONTRIBUTIONS

**Manouchehr Aghajanzadeh:** Conceptualization; data curation; formal analysis; funding acquisition; investigation; methodology; project administration; resources; software; supervision; validation; visualization; writing – original draft; writing – review and editing. **Ali Alavi Foumani:** Data curation; formal analysis; methodology; project administration; resources; supervision; validation; visualization; writing – original draft; writing – review and editing. **Azita Tangestaninejad:** Data curation; formal analysis; methodology; project administration; resources; validation; visualization; writing – original draft; writing – review and editing. **Mohammad Haghighi:** Data curation; methodology; project administration; resources; validation; visualization; writing – original draft; writing – review and editing. **Yousha Pourahmadi:** Data curation; formal analysis; methodology; project administration; resources; supervision; validation; visualization; writing – original draft; writing – review and editing. **Ehsan Hajipour Jafroudi:** Data curation; formal analysis; funding acquisition; investigation; methodology; project administration; resources; software; supervision; validation; visualization; writing – original draft; writing – review and editing. **Mahsa Mousazadeh:** Data curation; formal analysis; methodology; project administration; resources; supervision; validation; visualization; writing – original draft; writing – review and editing. **Rastin Hosseinzadeh Asli:** Data curation; formal analysis; methodology; project administration; resources; supervision; validation; visualization; writing – original draft; writing – review and editing.

## CONFLICT OF INTEREST STATEMENT

The authors report no conflict of interest.

## ETHICS STATEMENT

This case report was approved by ethic comity of Arya hospital and Department of Internal Medicine, Guilan University of Medical Sciences, Razi Hospital, Rasht, Iran.

## CONSENT

Written informed consent was obtained from the patient to publish this report in accordance with the journal's patient consent policy.

## Data Availability

Author elects to not share data.
